# Interpopulational Variations in Sexual Chemical Signals of Iberian Wall Lizards May Allow Maximizing Signal Efficiency under Different Climatic Conditions

**DOI:** 10.1371/journal.pone.0131492

**Published:** 2015-06-29

**Authors:** José Martín, Jesús Ortega, Pilar López

**Affiliations:** Departamento de Ecología Evolutiva, Museo Nacional de Ciencias Naturales, CSIC, José Gutiérrez Abascal 2, Madrid, Spain; Universidad de Granada, SPAIN

## Abstract

Sexual signals used in intraspecific communication are expected to evolve to maximize efficacy under a given climatic condition. Thus, chemical secretions of lizards might evolve in the evolutionary time to ensure that signals are perfectly tuned to local humidity and temperature conditions affecting their volatility and therefore their persistence and transmission through the environment. We tested experimentally whether interpopulational altitudinal differences in chemical composition of femoral gland secretions of male Iberian wall lizards (*Podarcis hispanicus*) have evolved to maximize efficacy of chemical signals in different environmental conditions. Chemical analyses first showed that the characteristics of chemical signals of male lizards differed between two populations inhabiting environments with different climatic conditions in spite of the fact that these two populations are closely related genetically. We also examined experimentally whether the temporal attenuation of the chemical stimuli depended on simulated climatic conditions. Thus, we used tongue-flick essays to test whether female lizards were able to detect male scent marks maintained under different conditions of temperature and humidity by chemosensory cues alone. Chemosensory tests showed that chemical signals of males had a lower efficacy (i.e. detectability and persistence) when temperature and dryness increase, but that these effects were more detrimental for signals of the highest elevation population, which occupies naturally colder and more humid environments. We suggest that the abiotic environment may cause a selective pressure on the form and expression of sexual chemical signals. Therefore, interpopulational differences in chemical profiles of femoral secretions of male *P*. *hispanicus* lizards may reflect adaptation to maximize the efficacy of the chemical signal in different climates.

## Introduction

Animals use sexual signals to inform conspecifics on different traits of the sender [1]. In many cases, these sexual signals evolve to ensure reliability of the message, and also to maximize signal efficacy in a given environment [1–3]. The efficacy of a signal depends on factors such as how well the signal is transmitted through the environment, durability or persistence of the signal, or how well the signal is detected by the sensory system of receivers. These factors will determine how selection shapes the characteristics of the signal in order to maximize perception by the receiver [2–5]. Darwinian selection may maximize the efficacy of sexual signals in different environments or under different climatic conditions [1, 5].

Chemoreception is one of the main sensory systems for many animals, including a number of vertebrates, and chemical signals play an important role in communication and sexual selection in these animals [6–8]. For example, in lizards, chemical signals from femoral glands or faeces are very often used to scent-mark substrates, allowing to delimit territories or attract mates [9–12]. Chemical signals in scent-marks may provide information on sex, size, dominance, and even details of health condition of the signaller [7, 13–16]. This information is important in intrasexual relationships between males [17–22] and in female mate choice [23–28]. Therefore, chemical signals are expected to evolve to honestly signal traits involved in sexual selection [16].

In addition, chemical signals used for scent marking should ensure efficiency by appropiately tuning to environmental factors such as local humidity and temperature (i.e. affecting their volatility and therefore their persistence and transmission through the environment) [4, 29–30]. Thus, it has been suggested that the observed interspecific or interpopulational differences in chemical signals of lizards might partly reflect selection for maximizing the efficacy of signals under different climatic conditions [4, 31–34]. However, this hypothesis has never been tested experimentally.

In this paper, we tested whether interpopulational differences in chemical composition of femoral gland secretions of male lizards may have evolved to maximize efficacy of chemical signals under different environmental conditions. We examined experimentally the effects of climatic conditions on the persistence and efficacy of chemical sexual signals of Iberian wall lizards (*Podarcis hispanicus*). This is a small lacertid lizard living in rocky habitats of the Iberian Peninsula. Males scent-mark substrates with femoral gland secretions, which contain a mixture of proteic and lipophilic compounds, mainly steroids, fatty acids, alcohols, and waxy esters [33–36]. Chemical signals are very important in intraspecific relationships of this lizard [37]. Males gauge scent-marks of other males to identify potential rivals and assess their competitive ability [17, 19–20]. Females show strong chemosensory responses and prefer areas scent-marked by males that allocate more cholesta-5,7-dien-3-ol to femoral secretions [23, 34, 36, 38], which are individuals of presumably high quality as suggested by, for example, their more efficient immune response [23].

This lizard species exhibits substantial levels of intraspecific phenotypic variation [39]. Molecular studies suggest that the Iberian wall lizard is paraphyletic and forms part of a species complex with at least five monophyletic lineages and several now well recognised species [39–41]. But even inside the same genetic lineages, there is an important phenotypic variation between some populations which could be due to the different environments that they occupy. For example, in Central Spain, there are several distinct populations of *P*. *hispanicus*, with clear phenotypic differences, that occupy nearby areas with very different climatic conditions [35, 42–43]. Moreover, the chemical composition of femoral secretions of males differs even between closely genetically related populations [36].

Here, we studied two genetically related populations of *P*. *hispanicus* lizards from the Guadarrama Mts. (Central Spain) that live at different altitudinal ranges with contrasting climatic differences. We 1) analysed chemical composition of femoral gland secretions of males of both populations using gas chromatography-mass spectrometry (GC-MS) and 2) tested the chemosensory ability of female lizards to detect males’ scent marks that were experimentally maintained under different temperature and humidity/dryness regimes. We examined whether the temporal attenuation of the chemical stimuli differed between populations depending on temperature and humidity. We predicted that the efficacy (i.e., detectability and persistence) of scent marks for each population should be maximal under the local environmental conditions of each population, and discussed how this local adaptation may be based on interpopulational differences in compounds in secretions.

## Material and Methods

### Study Animals

We captured adult *P*. *hispanicus* lizards by noosing during April-May 2012, at two populations at different elevations in the Guadarrama Mountains (Central Spain), separated 6 km by air. The ‘lowest elevation’ locality is an oak forest (`La Golondrina´) near Navacerrada village (40°43x N, 04°01´ W; 1,190 m altitude), where lizards can be found on granite rocky outcrops inside the forest. The ‘highest elevation’ locality is found in the upper part of `Fuenfría Valley´ near Cercedilla village (40°47´ N, 04°03´ W; 1,750 m altitude) occupying granite rock walls and rock piles at the edge of a pine forest. Analyses of microsatellite data showed a very low genetic divergence between these two populations and a high degree of gene flow, indicating that they belong to the same genetic lineage [36].

Lizards were taken to "El Ventorrillo" Field Station, about 5 km away from the sampling sites, the same day of capture. Lizards were kept in two walk-in climatic chambers (Ibercex V-450-D; ASL S.A., Madrid, Spain) where temperature (diurnal = 21 °C; nocturnal = 15 °C) and photoperiod (12 h:12 h, light:dark) were controlled automatically. Lizards were individually housed in plastic terraria (40 x 30 x 25 cm) filled with a moistened coconut fibre substrate and provided with a water bowl and a brick (24 x 11 x 8 cm) for shelter and as a basking platform. A 50 W halogen lamp was suspended over one end of the terrarium providing a diurnal temperature gradient (21–45 °C). This allowed thermoregulation of lizards around the preferred body temperature of this species (34.4°C) [44] while ensuring that lizards from each population could freely select different temperatures if there were any small population differences in thermoregulation requirements [42]. In addition, a fluorescent bulb over each shelf provided ambient lighting mimicking the natural photoperiod, and mercury vapour bulbs (Exoterra Solar Glow 125 W) provided ultraviolet radiation during 1.5 h a day (from 14.00 h to 15.30 h). Lizards were daily watered, and fed crickets (*Acheta domesticus*) and mealworms (*Tenebrio mollitor*), dusted with a commercial vitamin and calcium supplement, *ad libitum*.

All animals were healthy and were returned to their capture sites at the end of trials. Captures of lizards were performed under license (permit number: 10/072913.9/12) from the Environmental Agency of Madrid Government (“Consejería del Medio Ambiente de la Comunidad de Madrid”, Spain). Sampling procedures and experimental studies were reviewed and specifically approved as part of obtaining this field permit. The laboratory studies and the husbandry procedure were also approved by the Animal Ethics Committee of the Museo Nacional de Ciencias Naturales (CSIC).

### Climatic Conditions

We summarised the available environmental temperatures and precipitations in the study areas by using long-term (from 1978 to 2011) daily data from two nearby meteorological stations: ‘Colmenar Viejo’ (40°41’55” N, 03°45’52” W; elevation 1,004 m; Madrid province) and ‘Puerto de Navacerrada’ (40°46’50” N, 04°00’37” W; elevation 1,894 m; Madrid province) for the lowest and highest elevation population respectively (data available from the web of the Spanish Meteorological Agency, ‘Agencia Española de Metereología, AEMET’; *http://www.aemet.es*). Monthly temperatures were measured as means of daily mean temperatures, and we also calculated means of daily maximum air temperatures, as advised for ecophysiological studies of reptiles [45]. We also used total precipitation as an indication of humidity conditions. We used data from April to June, which coincides with the main mating season of lizards, when males have the highest rates of femoral secretions [33].

### Chemical Signals of Lizards

We extracted femoral gland secretion of male lizards from both populations by gently pressing around the femoral pores, and collected secretion directly in glass vials with glass inserts. We also used the same procedure on each sampling occasion, but without collecting secretion, to obtain blank control vials that were treated in the same manner to compare with the actual samples.

Samples of secretions were analyzed using a Finnigan-ThermoQuest Trace 2000 gas chromatograph (GC) fitted with a poly (5% diphenyl/ 95% dimethylsiloxane) column (Supelco, Equity-5, 30 m length x 0.25 mm ID, 0.25-μm film thickness) and a Finnigan-ThermoQuest Trace mass spectrometer (MS) as detector. Sample injections (2 μl of each sample dissolved in 200 μl of n-hexane; Sigma, capillary GC grade) were performed in splitless mode using helium as the carrier gas, with injector and detector temperatures at 270°C and 250°C, respectively. The oven temperature program was as follows: 50°C isothermal for 10 min, then increased to 280°C at a rate of 5°C/min, and then isothermal (280°C) for 20 min. Mass spectral fragments below m/z = 39 were not recorded. Impurities identified in the solvent and/or the control vial samples were not reported. Initial identification of secretion components was done by comparison of mass spectra in the NIST/EPA/NIH 2002 computerized mass spectral library. When possible, identifications were confirmed by comparison of spectra and retention times with those of authentic standards. Authentic samples were purchased from Sigma-Aldrich Chemical Co.

We identified and calculated relative proportions determined as the percent of the total ion current (TIC) of major compounds (> 1% of the TIC area) in secretions. To compare the compounds found in femoral secretions between the two populations, we used the compositional analysis, consisting of logit transforming the proportion data by taking the natural logarithm of proportion ⁄ (1 –proportion) to correct the problem of nonindependence of proportions [46]. Then, we calculated Euclidean distances between every pair of individual samples to produce a resemblance matrix that formed the basis of the analyses. We used single factor permutational multivariate analysis of variance tests (PERMANOVA) [47–48] based on the Euclidean resemblance matrix using 999 permutations to analyze whether the composition of the femoral secretions varied between populations and experimental conditions. Differences were investigated further using canonical analysis of principal coordinates (CAP) [49]. The software PRIMER V6.1.13 [50] with the PERMANOVA+ V1.0.3 add-on package [51] was used to investigate differences between chemical profiles.

### Chemosensory Trials

To test for the relative detectability, persistence and efficiency of femoral secretions of males, we designed an experiment to compare the chemosensory responses of female lizards in response to scent stimuli arising from cotton applicators bearing femoral secretions of males of their own populations. We especifically examined the effect of different conditions of temperature and humidity (see below) on the temporal fading of the chemical stimuli after the secretions had been deposited on the cotton swabs.

We prepared stimuli by taking out femoral secretions of males (*n* = 24; 12 from each population) pressing around the femoral pores and collected the waxy secretion directly on the cotton tip (1 cm) of a wooden applicator. We used approximately the same amount of femoral secretion in each stimulus (2×1 mm of solid secretion from each of three pores) to minimize the likelihood that differences in chemosensory responses were due to differences in the amount of secretion presented. To avoid differences in responses to different individual males [23], every female (*n* = 24; 12 from each population) was always tested with secretions from the same individual male from their same origin population. Secretions used for chemosensory detection tests came from different individual males than those used for chemical analyses (see above) due to limitations in the amount of secretion that a male can produce.

Immediately after, we placed swabs impregnated with chemical stimuli in two incubator chambers at 12°C and 22°C (‘cold’ vs. ‘warm’, respectively), and left them there for 1 min (initial time), 1 h or 3 h before being used in chemosensory tests. The cold and warm temperatures corresponded approximately to the mean of daily maximum temperatures (those experienced by lizards during their maximum diurnal peak of activity) in the highest and lowest elevation study areas respectively during the mating season. The cotton swabs were not in contact with anything inside the chambers, and after being used in a single test were discarded.

Each individual female (twelve from each population) was tested in six treatments: three periods of time since the femoral secretion was deposited x two temperatures, but participated in only one test every day in a random order. Trials were conducted in outdoor conditions during May and between 8:00–12:00 h (GMT) when lizards were fully active. Before the tests, females were allowed to bask and attain an optimal body temperature (around 34.4°C) [44]. In each trial, the same experimenter (PL), who was blind to the treatment, slowly approached a lizard’s home cage and slowly moved the cotton swab applicator attached to a long stick (50 cm) to a position 2 cm anterior to the lizard’s snout. Lizards allowed approaching and testing without fleeing. All female lizards responded to swabs by tongue flicking. Differential rates of tongue-flick (TF) of lizards to different chemical stimuli allow testing detection of chemical cues [52–54]. We recorded latency to the first TF as an indication of detectability (i.e. more detectable stimuli should have shorter latencies) and numbers of TFs directed to the swab during 1 min, beginning with the first TF, as an indication of the efficiency of the stimuli in eliciting chemosensory responses of females (i.e. more efficient stimuli should elicit higher TF rates because they are supposed to be more attractive) [25, 38, 54]. The comparison of responses to the femoral secretions freshly collected from males, and responses to secretions that had spent some time exposed to the environment climatic conditions after they were deposited, allowed testing for temporal persistence of the chemical stimuli in secretions.

In a second experiment, we examined the effect of dryness on the temporal fading of the chemical stimuli after they had been deposited on the cotton swabs under different humidity conditions. We followed a procedure similar to the previous experiment but cotton swabs with femoral secretions of males (*n* = 24; different individuals than in previous tests) were placed in hermetic glass boxes containing either a 2 cm depth water substrate (‘humid’ treatment), which resulted in a water saturated air inside the box, or silica gel (‘dry’ treatment), which absorbed humidity and provided a dry environment, during 1 min, 1 h or 3 h. The boxes were placed indoor, protected from light and at the room environment temperature (about 15–17°C). Chemosensory tests were made as in the previous experiment but using different individual females (*n* = 24; 12 from each population).

To examine differences in latencies or number of directed TFs of the same individual females among treatments, we used repeated measures General Linear Models (LMs) with population (lower *vs*. higher elevation) as a between factor, and time (initial *vs*. 1 h *vs*. 3 h) and temperature (cold *vs*. warm) or dryness treatment (dry *vs*. humid) as within-subjects factors, including the interactions in the models. Data were log-transformed to ensure normality (Shapiro-Wilk’s test). Tests of homogeneity of variances (Levene's test) showed that in all cases, variances were not significantly heterogeneous after transformation. Pairwise comparisons used Tukey’s honestly significant difference tests [55]. All the statistical analyses were performed with STATISTICA v8.0 (Statsoft Inc., Tulsa, OK, USA).

## Results

### Climatic Conditions

**Fig 1:**
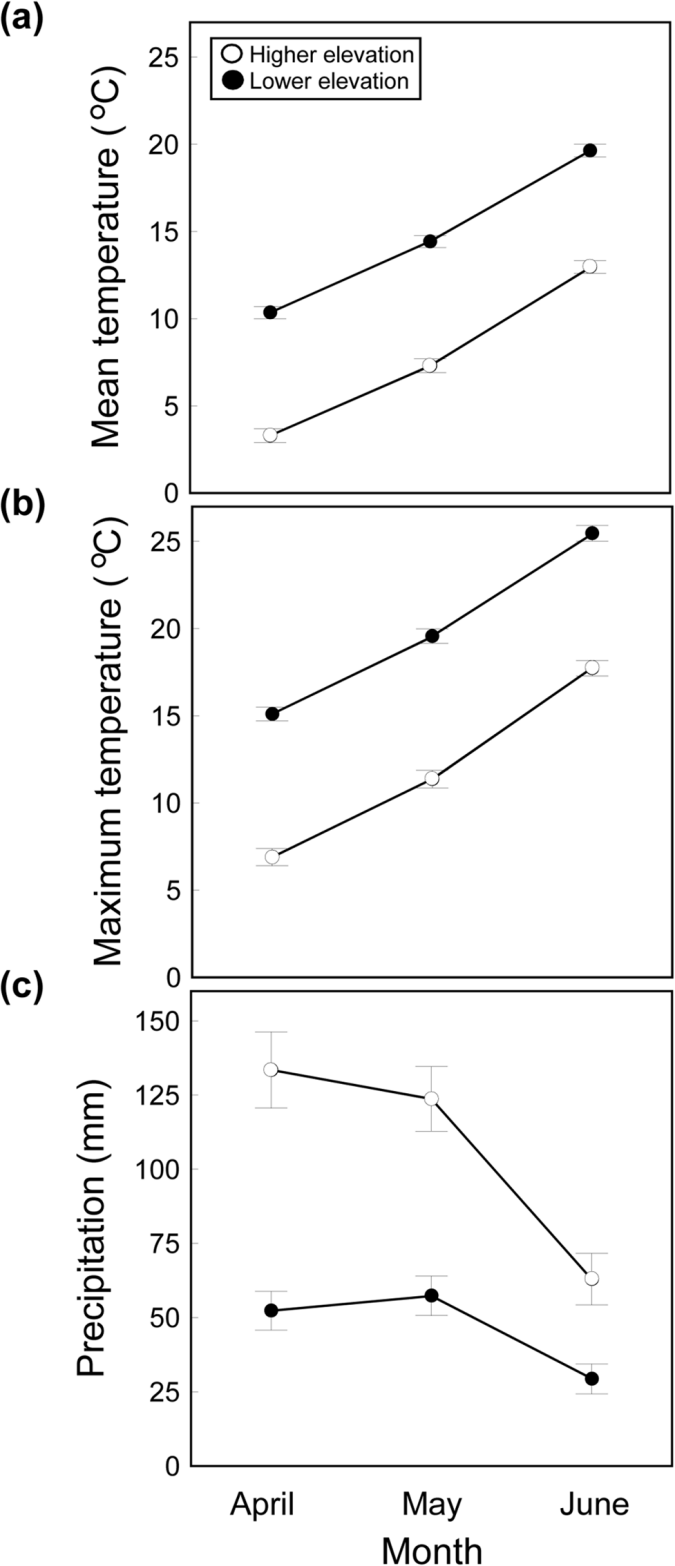
Climatic Conditions at the Lizards’ Populations. Variation in (a) daily mean and (b) daily maximum air temperatures, and (c) total monthly precipitation in two populations (lower elevation: black circles; higher elevation: grey circles) of the Guadarrama Mts., Central Spain, in the months of April, May and June, coinciding with the mating season of *Podarcis hispanicus* lizards. Data show average (± SE) monthly values for a 34 years period (1978–2011).

The mean air temperatures increased significantly within a given year from April to June in both populations but temperatures were significantly greater in the lowest elevation population in all these months (two-way ANOVA; month: *F*_2,193_ = 328.83, *p* < 0.0001, partial *η* ^*2*^ = 0.77; population: *F*_1,193_ = 523.62, *p* < 0.0001, partial *η* ^*2*^ = 0.73; interaction: *F*_2,193_ = 0.22, *p* = 0.80, partial *η* ^*2*^ = 0.002) (Fig 1A). A similar result was observed for the means of daily maximum air temperatures (two-way ANOVA; month: *F*_2,193_ = 276·02, *p* < 0.0001, partial *η* ^*2*^ = 0.74; population: *F*_1,193_ = 471.91, *p* < 0.0001, partial *η* ^*2*^ = 0.71; interaction: *F*_2,193_ = 0.18, *p* = 0.83, partial *η* ^*2*^ = 0.002) (Fig 1B). Total precipitation decreased significantly from April to June in both populations and precipitations were significantly greater in the highest elevation population in April and May, but not in June (two-way ANOVA; month: *F*_2,193_ = 17.25, *p* < 0.0001, partial *η* ^*2*^ = 0.15; population: *F*_1,193_ = 67.71, *p* < 0.0001, partial *η* ^*2*^ = 0.26; interaction: *F*_2,193_ = 3.67, *p* = 0.0027, partial *η* ^*2*^ = 0.04) (Fig 1C). Therefore, the highest elevation population has a climate that is colder and more humid than in the lowest elevation population.

### Chemical Signals of Lizards

Femoral secretions of males from the two populations were formed by similar types of compounds (S1 Table), mainly steroids (83.88%, both populations combined), but also fatty acids between C_8_ and C_24_ (10.95%), waxy esters (2.24%), alcohols (1.53%), an aldehyde (0.67%), terpenoids (0.65%), a ketone (0.06%), and a furanone (0.02%). Considering specific compounds, cholesterol (56.2%) was the most abundant one, followed by cholesta-5,7-dien-3-ol (15.9%), hexadecanoic acid (3.0%), octadecenoic acid (2.8%), and ergosta-5,8-dien-3-ol (2.8%). Both populations shared 50 out of the 61 detected compounds (82%) including all the main ones (i.e. those with proportions > 0.5%). However, there were clear interpopulational differences in proportion of compounds. Thus, the PERMANOVA based on the resemblance matrix comparing the chemical profiles of males from the two populations was statistically significant (pseudo *F*_1,45_ = 13.65, *p* < 0.001). The CAP analysis classified 91.5% of the individual chemical profiles into the correct population using leave-one-out cross-validation and *m* = 4 axes (*δ*_1_^2^ = 0.65, *p* = 0.001). Comparing specific types of compounds, lizards from the lowest elevation population had significantly higher proportions of cholesterol (LM; *F*_1,45_ = 16.43, *p* = 0.0002) and fatty acids (*F*_1,45_ = 12.99, *p* < 0.0008) but significantly lower proportions of alcohols (*F*_1,45_ = 6.09, *p* = 0.017) than lizards from the highest elevation population. However, there were not significant differences between populations in proportions of cholesta-5.7-dien-3-ol (*F*_1,45_ = 0.06, *p* = 0.80) or other types of compounds, such as waxy esters or terpenoids (*p* > 0.30 in all cases).

### Effects of Temperature on Signal Efficacy

**Fig 2:**
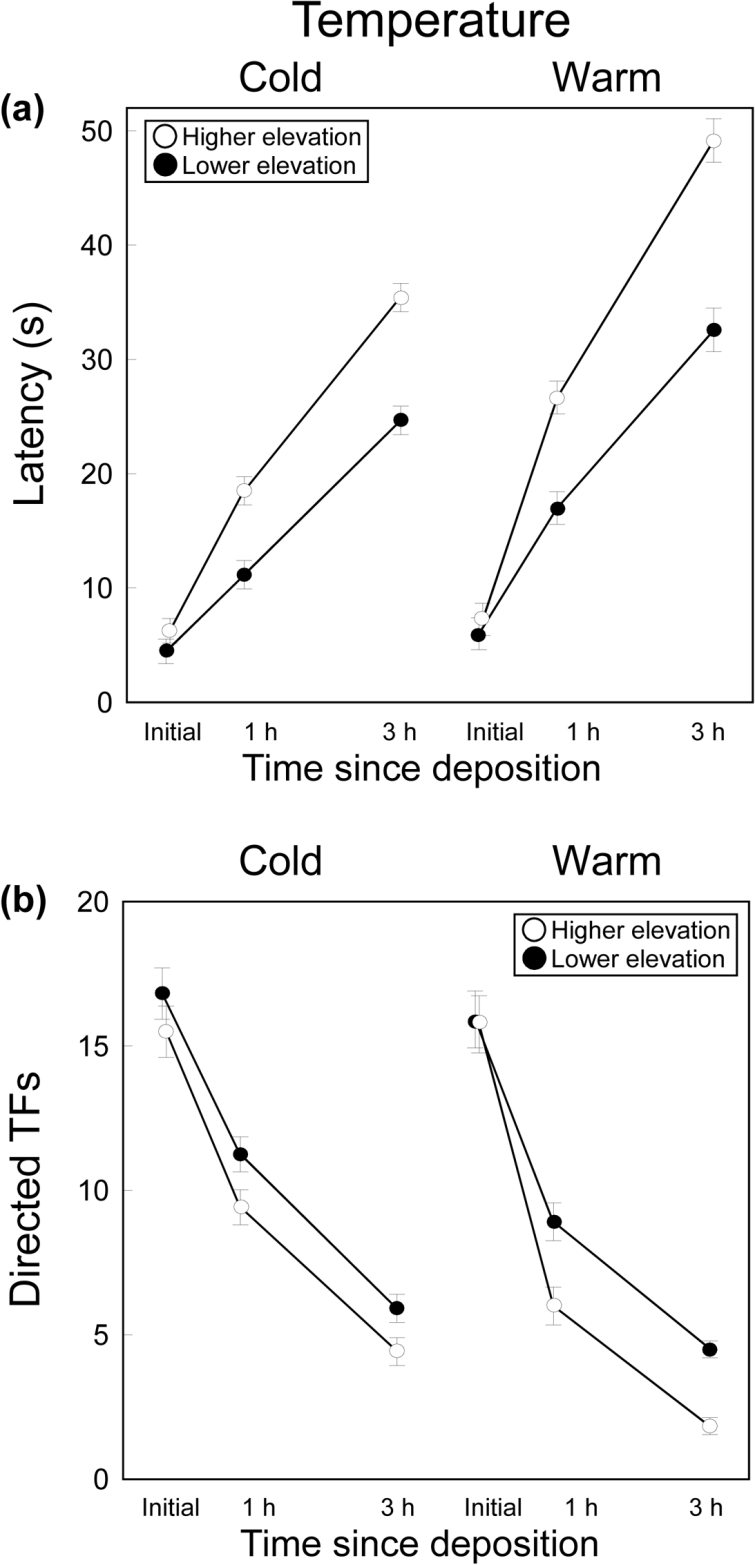
Effects of Temperature on Signal Efficacy. Mean ± SE (a) latency and (b) number of tongue-flicks (TF) directed to swabs by female *P*. *hispanicus* lizards from two populations (lower elevation: black; higher elevation: white) in response to swabs bearing femoral secretions of males immediately after they were secreted (‘initial’), 1 h or 3 h since deposition and maintained under two temperature regimes (cold *vs*. warm).

The latency to the first TF differed significantly between populations and temperatures and among time periods, and all the two-way interactions were significant (Table 1; Fig 2A). Females had longer latencies as time since the secretions were deposited increases, and when secretions were previously exposed to warmer temperatures. Therefore, the detectability of the signal decreased with time and at warmer temperatures in both populations. However, the temperature*population and time*population significant interactions showed that these effects (i.e. the greater loss of detectability with time at warm temperature) were more marked in the highest elevation population, where local temperatures were cold. Thus, initial latencies did not significantly differ between populations nor between treatments (Tukey’s tests, *p* > 0.96 in all cases), but latencies 1h and 3 h after deposition were significantly longer in the warm than in the cold treatment in both populations (lower elevation: *p* = 0.001; higher elevation: *p* = 0.0001), and, within each temperature treatment, latencies were significantly longer in the highest than in the lowest elevation population (*p* = 0.0001 in all cases) (Fig 2A).

**Table 1:** Statistical Tests for the Effects of Temperature and Dryness on Signal Efficacy.

		Latency			Tongue-flicks		
	df	*F*	*p*	partial *η* ^*2*^	*F*	*p*	partial *η* ^*2*^
**Effects of temperature:**							
** Population**	1,22	50.85	<0.0001	0.70	8.19	<0.01	0.27
** Temperature**	1,22	118.83	<0.0001	0.84	50.33	<0.0001	0.70
** Time**	2,44	390.87	<0.0001	0.95	360.58	<0.0001	0.94
** Temperature x Population**	1,22	4.72	0.041	0.18	0.01	0.95	0.0002
** Time x Population**	2,44	16.56	<0.0001	0.43	0.84	0.44	0.04
** Temperature x Time**	2,44	17.22	<0.0001	0.44	4.07	0.02	0.16
** Population x Temperature x Time**	2,44	1.90	0.16	0.08	3.44	0.04	0.13
**Effects of dryness:**							
** Population**	1,22	15.93	<0.0001	0.42	13.51	0.0013	0.38
** Dryness**	1,22	142.38	<0.0001	0.87	52.20	<0.0001	0.70
** Time**	2,44	431.63	<0.0001	0.95	256.21	<0.0001	0.92
** Dryness x Population**	1,22	15.82	0.0006	0.42	9.04	0.0065	0.29
** Time x Population**	2,44	4.01	0.025	0.15	2.85	0.07	0.11
** Dryness x Time**	2,44	24.48	<0.0001	0.53	4.84	0.013	0.18
** Population x Dryness x Time**	2,44	4.80	0.013	0.18	0.63	0.54	0.03

Results of full factorial General Linear Models (LMs) examining variation in latencies or number of directed tongue-flicks of the same individual female *P*. *hispanicus* lizard among treatments, with population (lowler *vs*. higher elevation) as a between factor, and temperature (cold *vs*. warm) or dryness (dry *vs*. humid) treatments and time since deposition (initial *vs*. 1 h *vs*. 3 h) as within-subjects factors. Degrees of freedom (df), *F* statistics, significance levels (*p*) and effect sizes (partial *η* ^2^) are shown.

The number of TFs directed by females differed significantly between populations and temperatures, and among time periods, and the temperature*time interaction was significant (Table 1; Fig 2B). Thus, in both populations, TF rates of females decreased as the time since the secretion was deposited increased and when secretions were previously exposed to warmer temperatures. Therefore, the signal elicited lower responses (was less effective) when time and temperature increased. However, the population* temperature*time triple significant interaction indicated that the combined detrimental effects of temperature and time were more marked in the highest elevation population. Thus, while initial TF rates did not significantly differ between populations (Tukey’s tests, *p* > 0.67 in all cases), TF rates 1 and 3 h after deposition did not significantly differ between populations in the cold treatment (*p* > 0.87 in both cases), but in the warm treatment, TF rates 1 and 3 h after deposition were significantly lower in the highest elevation population (*p* < 0.05 in both cases) (Fig 2B).

### Effects of Dryness on Signal Efficacy

**Fig 3:**
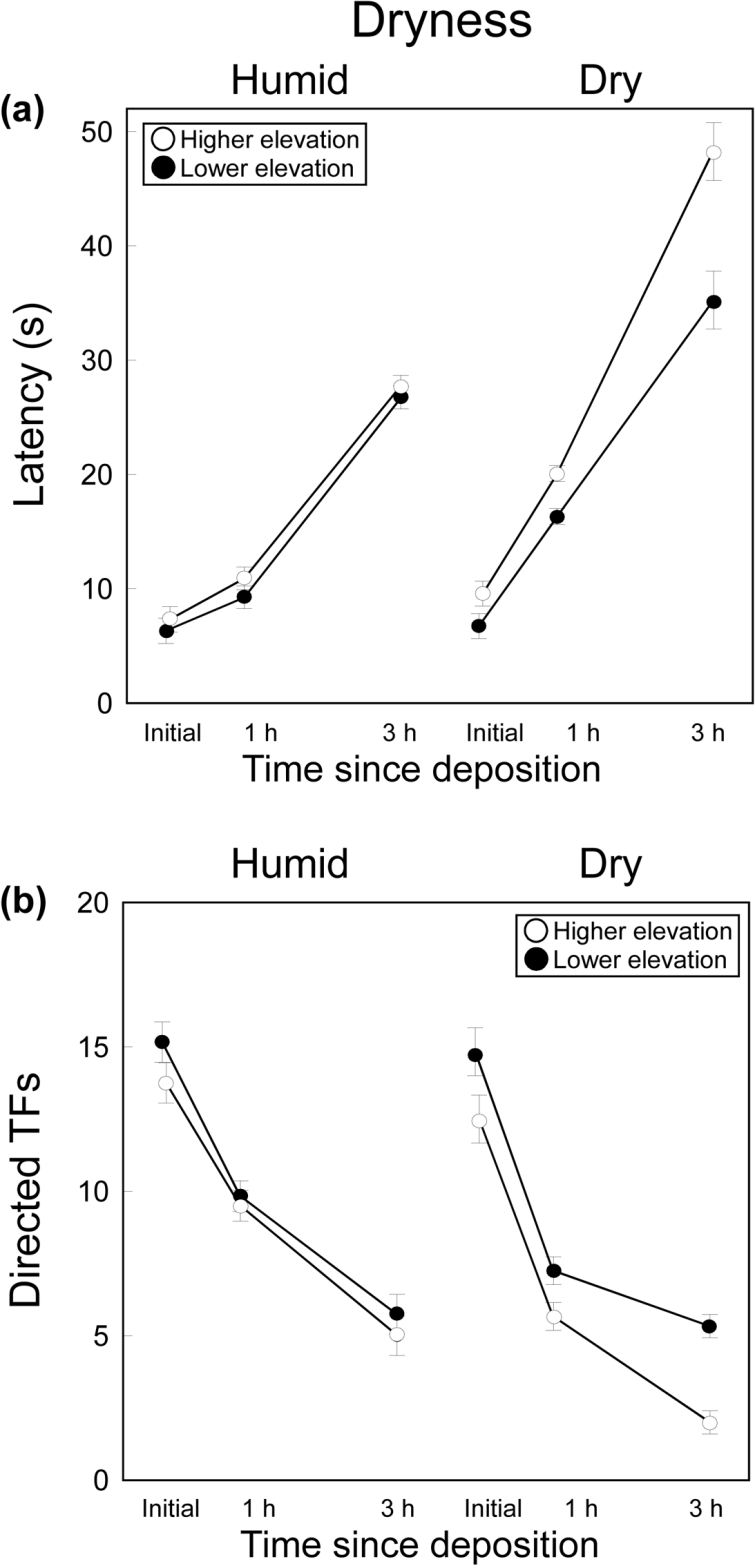
Effects of Dryness on Signal Efficacy. Mean ± SE (a) latency and (b) number of tongue-flicks (TF) directed to swabs by female *P*. *hispanicus* lizards from two populations (lower elevation: black; higher elevation: white) in response to swabs bearing femoral secretions of males immediately after they were secreted (‘initial’), 1 h or 3 h since deposition and maintained under two dryness conditions (humid *vs*. dry).

The latency to the first TF differed significantly between populations and dryness treatments and among time periods, and all the interactions were significant (Table 1; Fig 3A). Therefore, females in both populations had significantly longer latencies (i.e. the signal had lower detectability) as the time since the secretion was deposited increased and when secretions were previously exposed to drier conditions. However, the significant interactions showed that these effects (i.e. the greater loss of detectability with time under drier conditions) were more marked in the highest elevation population, where the local climate was more humid. Thus, initial latencies did not differ significantly between populations nor between temperature treatments (Tukey’s tests, *p* > 0.96 in all cases), latencies 1 h and 3 h after deposition did not differ between populations in the humid treatment (*p* > 0.99 in both cases), but in the dry treatment latencies were significantly longer in the highest elevation than in the lowest elevation population (*p* < 0.001 in both cases) (Fig 3A).

The number of TFs directed by females differed significantly between populations and dryness treatments, and among time periods, and dryness*population and dryness*time interactions were significant (Table 1; Fig 3B). Thus, in both populations, TF rates of females decreased as the time since the secretion was deposited increased and when secretions were previously exposed to drier conditions. Therefore, the signal elicited lower responses (i.e. was less effective) as time and dryness increased. However, the significant interactions indicated that the detrimental effect of dryness was more marked in the highest elevation population. Thus, while initial TF rates did not significantly differ between populations nor between dryness treatments (Tukey’s tests, *p* > 0.07 in all cases), TF rates 1 and 3 h after deposition did not significantly differ between dryness treatments in the lowest elevation population (1h: *p* > 0.07; 3 h: *p* = 0.99) but TF rates were significantly lower in the dryness treatment in the highest elevation population (1h: *p* < 0.001; 3 h: *p* = 0.018) (Fig 3B).

## Discussion

Our results showed that the characteristics of chemical signals of male *P*. *hispanicus* lizards differed between two populations inhabiting environments with different climatic conditions in spite of the fact that these two populations are closely related genetically [36]. Moreover, chemosensory tests with female lizards showed that chemical signals of males had a lower efficacy (i.e. detectability and persistence) when temperature and dryness increase, but that these effects were more detrimental in the highest elevation population, which occupies naturally colder and more humid environments. Therefore, we suggest that interpopulational differences in chemical profiles of femoral secretions of male *P*. *hispanicus* lizards may reflect adaptation to maximize the efficacy of the chemical signal in different climates. Nevertheless, our study suffers the limitation of considering only two populations, and, to confirm this hypothesis, further studies should include more populations reflecting a richer cline in climatic conditions.

### Climatic Conditions and Chemical Signals Composition

Chemical analyses confirmed interpopulational differences in chemical composition of femoral secretions of male *P*. *hispanicus* lizards, as among other populations [33–36, 56]. These differences occur not only among genetically distinct populations or species in the *P*. *hispanicus* species complex [35, 56], which might be simply explained by genetic drift without invoking adaptive reasons, but also among populations that are closely related genetically [36]. The latter suggests that local adaptation to different environments may drive differences in chemical signals.

The results of this and previous studies showed that differences in chemical composition of femoral secretions between these two *P*. *hispanicus* lizard populations are due to lower elevation males having higher proportions of cholesterol and fatty acids, and lower proportions of alcohols than higher elevation males. These different compositions should result in different physical-chemical properties of the entire femoral secretion under different temperature and humidity conditions. Thus, under the naturally higher temperatures and drier conditions of the lowest elevation population, the higher relative proportion of cholesterol (the main compound in secretions) may contribute to avoid degradation of other more easily alterable compounds that are known to be important in intraspecific communication in this lizard such as cholesta-5,7-dien-3-ol [23, 34, 38], which, however, did not differ between populations. It was already suggested that cholesterol, the major compound in secretions of many lizard species [57], might not have a signalling function, but just form a matrix that could protect other compounds, which would be the true semiochemicals [32].

Female *P*. *hispanicus* show strong chemosensory responses to both cholesterol and cholesta-5,7-dien-3-ol (but responses are higher to the latter) [38]. Females might use the abundant cholesterol to detect, via chemosensory senses alone, the substrate scent marks from males, and later use proportions of the less abundant cholesta-5,7-dien-3-ol to evaluate the quality of the male. Experimental studies indicated that female *P*. *hispanicus* do not prefer scents marks of males with higher proportions of cholesterol, but prefer marks with higher proportions of cholesta-5,7-dien-3-ol [23]. This steroid is a precursor of vitamin D_3_, which is essential in calcium metabolism and a potent immuno-stimulator [58]. Thus, there could be a potential conflict between allocating high proportions of this steroid to femoral secretions and maintaining simultaneously an appropriate immune response. This trade-off, which only genuinely high quality males may afford [59], would confer honesty to the chemical signal [16].

Similarly, the greater abundance of alcohols in secretions of the highest elevation population would not be useful under higher temperatures where alcohols will evaporate more quickly, which may explain why lower elevation males allocated lower proportions of alcohols to secretions. The function of alcohols in secretions is not known, but in other lacertid species, alcohols are related to the social status of a male (i.e. more dominant males have higher proportions of some alcohols in secretions) eliciting in other males aggressive responses [22, 60]. A previous study showed that male *P*. *hispanicus* from the lowest elevation population showed lower chemosensory responses to alcohols than males from the highest elevation population, suggesting interpopulational differences in relative importance of alcohols in communication [61]. These differences might be linked to the different efficacy of alcohols in scent marks under different climatic conditions.

Interpopulational differences in chemical composition may be explained not only by local adaptation but also by developmental plasticity as a direct consequence of the environmental thermal conditions experienced by lizards in each population. Thus, an experimental study shows that differences in basking conditions in the laboratory cause plastic changes in the composition of femoral secretions of male lizards *Podarcis muralis* [62]. This suggests that total time spent at optimal body temperatures may affect average metabolic rates and general health of lizards, which would result in a modification of their condition-dependent chemical signals [16]. Similarly, in our study, the observed interpopulational differences in chemical profiles could reflect the long-term effect of having been exposed to their respective native climatic conditions. Nevertheless, in spite of differences in climatic conditions, which do affect directly deposited scent marks, lizards could be able to obtain similar thermoregulatory precision in both populations through flexible thermoregulatory behaviour [42]. A further crossed experimental study (population of origin x thermal conditions of scent mark donors) would be necessary to disentangle local adaptation *vs*. developmental plasticity effects. However, both processes might result in a higher efficiency of chemical signals under local climatic conditions.

### Effects of Climatic Conditions on Signal Efficacy

With respect to the chemosensory experiments, our results indicate that females detected later (i.e. they had longer latency times) and had lower chemosensory tongue-flick responses to the femoral secretions of males as the time since deposition increased. This indicated that the chemical stimuli in secretions faded with time, very likely because chemical compounds that elicit responses evaporated and degraded with time since they were secreted [63]. In addition, the loss of detectability and efficacy of the chemical signal was faster under warm temperature and under drier conditions. This is because high temperatures increase evaporation and diffusion rates of chemicals, affecting their persistence [29–30]. Detrimental effects of higher temperatures on the efficacy of scent-marks were found in another lacertid lizard species [64]. Similarly, high temperatures limit trail-following behaviour of ants by accelerating pheromone decays [65–66]. High levels of humidity also increase evaporation and may oxidate some compounds [29–30]. However, we have found that dryness may have more negative effects on scent marks of *P*. *hispanicus* lizards than humidity. This may be explained because considering the normal climatic conditions experienced by these lizards during the mating season, scent-marks of lizards of both populations might be adapted to some moderate levels of humidity, but not to unusual high levels of dryness, which might alter the chemical structure of some relevant compounds.

Interestingly, these detrimental effects of higher temperatures and drier conditions affected differentially secretions of the two populations. Thus, secretions from the lowest elevation population, which occupies warmer and drier environments, seem to be less affected by these simulated conditions that resembled local ones. In contrast, secretions from the highest elevation population, which occupies fresher and more humid environments, suffered a quicker degradation of efficiency under climatic conditions similar to those of the lowest elevation population. These results support that characteristics of femoral secretions of males are adapted to local conditions of temperature and humidity in order to improve their efficiency. Interpopulational differences in the properties of compounds found in the chemical profiles also support this conclusion. Alternatively, further studies should consider whether the observed differences in chemosensory responses might be explained because the vomeronasal systems of females from each population might also have coevolved to be more efficient in detecting scent marks of males under the local environmental conditions.

In addition to changes in the chemical composition of secretions, the effects of different environmental conditions on the persistence of scent marks might also be compensated by increasing the amount of secretion produced. Mechanisms can be, for example, increasing the number or size of femoral glands in warmer climates to compensate for a quicker evaporation [31, 67]. Nevertheless, this strategy alone (i.e. without additionally modifying compounds in secretions), might not be enough to compensate for different climates as interspecific variation in the number of femoral pores in lacertid lizards seemed independent on climatic variables [68]. Also, when unfavourable environmental conditions render chemical signals very costly or not useful, an alternative communication system (i.e. visual) may be favoured [69].

### Consequences of Local Adaptation of Chemical Signals

What are the consequences of these microgeographic differences in male chemical signals in *P*. *hispanicus*? If variation in male mating signals to maximize efficacy in local conditions co-evolve with female preferences for the characteristics of the signal of the males of their own population, reproductive isolation and divergence between populations might arise as a consequence [34–35]. This could contribute to explain, at least partially, the genetic divergence observed between populations that inhabit contrasted environments in this lizard species complex [40]. Nevertheless, females may use some characteristics, or compounds, of the chemical signal that do not vary between populations in sexual selection [36], ignoring other differing compounds. This is because these characteristics may represent the strategic design of the signal, informing honestly on the quality of a male [16]. Interpopulational differences in chemical signals might only be aimed to protect and maximize the persistence of the compounds that convey the true message, which would be the same in the two populations. Therefore, this might prevent genetic divergence between these populations in spite of differences in chemical signals. Finally, from a conservationist point of view, our study suggests that if climatic conditions (temperature and humidity) change quickly due to global warming, this could decrease the efficacy of chemical sexual signals of wall lizards. This might disrupt sexual selection processes [64], affecting survivorship of populations.

## Supporting Information

S1 Table Lipophilic compounds found in femoral gland secretions of male lizards, *P*. *hispanicus* from a lower and a higher elevation population of the Guadarrama Mountains (Madrid, Spain). The relative amount of each component was determined as the percent of the total ion current (TIC) and reported as the average (±1SE). Characteristics (m/z) are reported for some unidentified (Un.) compounds.
(DOC)
