# Supplementary material for: Interpopulational Variations in Sexual Chemical Signals of Iberian Wall Lizards May Allow Maximizing Signal Efficiency under Different Climatic Conditions
Source: PLoS One. 2015 Jun 29;10(6):e0131492. doi: 10.1371/journal.pone.0131492 (PMC4488078; doi:10.1371/journal.pone.0131492)
Supplement: S1 Table — The relative amount of each component was determined as the percent of the total ion current (TIC) and reported as the average (±1SE). Characteristics (m/z) are reported for some unidentified (Un.) compounds. (DOC) [file pone.0131492.s001.doc]

**S1 Table**

|  |  | Populations | | | | | | |
| --- | --- | --- | --- | --- | --- | --- | --- | --- |
| RT | Compound | Lower elevation  (*n* = 24) | | | Higher elevation  (*n* = 23) | | | |
| Fatty acids: | |  | | |  | | | |
| 22.3 | Octanoic acid | 0.08 | + | 0.06 | 0.02 | + | 0.01 |  |
| 25.2 | Nonanoic acid | 0.32 | + | 0.15 | 0.20 | + | 0.10 |  |
| 27.9 | Decanoic acid | 0.08 | + | 0.03 | 0.03 | + | 0.02 |  |
| 32.9 | Dodecanoic acid | 2.18 | + | 0.92 | 1.29 | + | 0.63 |  |
| 34.4 | Dodecanoic acid, 1-methylethyl ester | 0.04 | + | 0.06 | 0.01 | + | 0.02 |  |
| 37.3 | Tetradecanoic acid | 0.02 | + | 0.02 | 0.01 | + | 0.02 |  |
| 39.4 | Pentadecanoic acid | 0.01 | + | 0.02 | 0.01 | + | 0.02 |  |
| 41.1 | Hexadecenoic acid | 0.12 | + | 0.03 | 0.09 | + | 0.08 |  |
| 41.5 | Hexadecanoic acid | 3.06 | + | 1.30 | 2.98 | + | 2.44 |  |
| 43.3 | Heptadecanoic acid | 0.01 | + | 0.02 | 0.02 | + | 0.02 |  |
| 44.7 | 9,12-Octadecadienoic acid | 1.14 | + | 0.56 | 0.68 | + | 0.88 |  |
| 44.8 | Octadecenoic acid | 2.83 | + | 1.08 | 2.68 | + | 1.83 |  |
| 45.2 | Octadecanoic acid | 0.87 | + | 0.28 | 0.94 | + | 0.70 |  |
| 47.6 | 5,8,11,14-Eicosatetraenoic acid, ethyl ester | 0.15 | + | 0.07 | 0.13 | + | 0.22 |  |
| 48.2 | Erucic acid | 0.06 | + | 0.06 | 0.08 | + | 0.05 |  |
| 48.6 | Eicosanoic acid | 0.23 | + | 0.13 | 0.58 | + | 0.45 |  |
| 50.3 | Heneicosanoic acid |  | - |  | 0.02 | + | 0.03 |  |
| 51.8 | Docosanoic acid | 0.25 | + | 0.09 | 0.63 | + | 0.44 |  |
| 54.8 | Tetracosanoic acid |  | - |  | 0.03 | + | 0.06 |  |
| Steroids: | |  |  |  |  |  |  |  |
| 56.5 | Cholest-2-4-diene | 0.36 | + | 0.08 | 0.58 | + | 0.34 |  |
| 56.8 | Cholesta-3.5-diene | 0.02 | + | 0.02 | 0.07 | + | 0.05 |  |
| 57.2 | Un. steroid (155,197,251,350,365) | 0.75 | + | 0.27 | 1.28 | + | 0.75 |  |
| 57.4 | Cholesta-5,7,9(11)-trien-3-ol | 0.33 | + | 0.15 | 0.62 | + | 0.40 |  |
| 57.6 | Un. steroid (207,251,350,365) | 0.11 | + | 0.05 | 0.33 | + | 0.09 |  |
| 57.7 | Un. steroid (143,195,207,351,366) | 0.11 | + | 0.04 | 0.37 | + | 0.09 |  |
| 58.5 | Un. steroid (141,156,209,350,365) |  | - |  | 0.05 | + | 0.04 |  |
| 58.8 | Un. steroid (155,197,251,365,379) |  | - |  | 0.20 | + | 0.13 |  |
| 59.2 | Cholesta-4,6-dien-3-ol | 0.04 | + | 0.04 | 0.17 | + | 0.22 |  |
| 60.0 | Un. steroid (195,209,251,365,379) | 0.04 | + | 0.06 | 0.07 | + | 0.07 |  |
| 60.9 | Cholesterol | 60.66 | + | 3.85 | 51.66 | + | 3.12 |  |
| 61.0 | Cholestanol | 0.60 | + | 0.78 | 0.46 | + | 0.96 |  |
| 61.7 | Cholesta-5.7-dien-3-ol | 14.62 | + | 3.99 | 19.19 | + | 2.95 |  |
| 63.2 | Un. steroid (105,213,353,368,386,415) | 0.05 | + | 0.08 | 0.08 | + | 0.07 |  |
| 63.3 | Campesterol | 0.45 | + | 0.14 | 0.33 | + | 0.21 |  |
| 64.4 | Ergosta-5,8-dien-3-ol | 2.32 | + | 0.35 | 3.34 | + | 0.42 |  |
| 64.9 | Cholesta-4,6-dien-3-one | 0.16 | + | 0.17 | 0.83 | + | 0.48 |  |
| 65.9 | Sitosterol | 0.02 | + | 0.04 | 0.03 | + | 0.07 |  |
| 66.4 | Un. steroid (214,267,365,380) | 2.14 | + | 1.38 | 1.86 | + | 0.62 |  |
| 67.3 | 4,4-Dimethyl-cholesta-5,7-dien-3-ol- | 0.99 | + | 0.62 | 2.02 | + | 0.52 |  |
| 67.7 | Stigmasterol |  | - |  | 0.02 | + | 0.04 |  |
| 73.9 | Cholest-5-en-3-ol, acetate |  | - |  | 0.43 | + | 0.39 |  |
| Alcohols: | |  |  |  |  |  |  |  |
| 35.9 | Tetradecanol | 0.26 | + | 0.15 | 0.42 | + | 0.27 |  |
| 40.1 | Hexadecanol | 0.03 | + | 0.03 | 0.04 | + | 0.03 |  |
| 43.8 | Octadecanol | 0.03 | + | 0.03 | 0.12 | + | 0.09 |  |
| 47.4 | Eicosanol | 0.09 | + | 0.06 | 0.46 | + | 0.37 |  |
| 50.7 | Docosanol | 0.15 | + | 0.07 | 0.44 | + | 0.34 |  |
| 53.8 | Tetracosanol | 0.17 | + | 0.02 | 0.46 | + | 0.22 |  |
| 56.6 | Hexacosanol | 0.11 | + | 0.04 | 0.30 | + | 0.16 |  |
| Terpenoids: | |  |  |  |  |  |  |  |
| 55.7 | Squalene | 0.40 | + | 0.11 | 0.44 | + | 0.37 |  |
| 56.1 | Unidentified terpenoid 1 | 0.06 | + | 0.06 | 0.14 | + | 0.11 |  |
| 60.5 | Unidentified terpenoid 2 |  | - |  | 0.26 | + | 0.18 |  |
| Waxy esters: | |  |  |  |  |  |  |  |
| 53.2 | Tetradecyl hexadecanoate |  | - |  | 0.02 | + | 0.04 |  |
| 63.5 | Octadecenyl octadecenoate |  | - |  | 0.13 | + | 0.28 |  |
| 65.2 | Hexadecyl hexadecanoate |  | - |  | 0.18 | + | 0.38 |  |
| 66.7 | Tetradecyl octadecenoate | 1.37 | + | 4.80 | 0.01 | + | 0.02 |  |
| 70.3 | Octadecyl hexadecanoate | 0.74 | + | 0.98 | 0.23 | + | 0.42 |  |
| 75.5 | Octadecyl octadecenoate | 0.37 | + | 0.79 | 1.37 | + | 2.18 |  |
| 76.0 | Eicosyl hexadecenoate |  | - |  | 0.05 | + | 0.11 |  |
| Others: | |  |  |  |  |  |  |  |
| 19.8 | Nonanal | 0.92 | + | 0.52 | 0.43 | + | 0.42 |  |
| 40.3 | Nonadecanone | 0.04 | + | 0.02 | 0.08 | + | 0.03 |  |
| 44.2 | Unidentified furanone | 0.01 | + | 0.02 | 0.03 | + | 0.03 |  |
